# Ronaldo Santos do Amaral (★1945 †2020)

**DOI:** 10.1590/0037-8682-0222-2020

**Published:** 2020-08-26

**Authors:** Pedro Luiz Tauil

**Affiliations:** 1 Universidade de Brasília, Núcleo de Medicina Tropical, Brasília, DF, Brasil.

On March 15, 2020, after a life of 74 years, the Sanitary Medical Doctor Ronaldo Santos do Amaral died in Brasilia, Federal District, succumbing after a long battle in a fight against a severe chronic disease. He was born in Salvador, Bahia, on November 22, 1945. Although he came from a simple and hardworking family, he aspired to become a medical doctor despite a very difficult social and economic context. In the mid-1960s, he succeeded in entering the Faculty of Medicine of Bahia University and graduated in the beginning of 1970s. After a brief stint as clinical doctor in small cities in the interior of Bahia, including São Francisco do Conde-his parents’ homeland-he embarked on what would ultimately become his great mission-Public Health.

**Figure:**
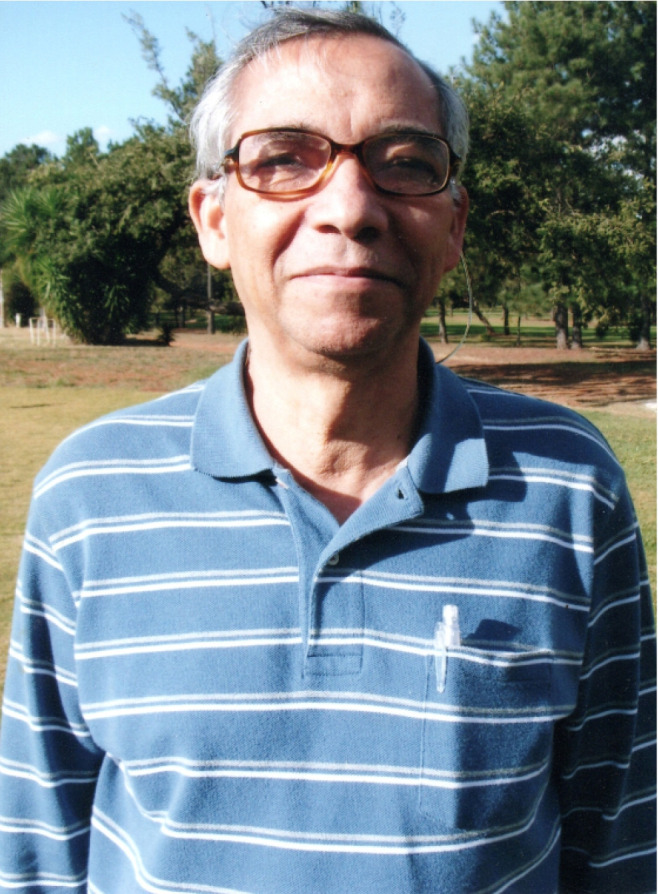

In 1973, he joined the former Fundação de Serviços de Saúde Pública (FSESP), and soon after, the former Superintendência de Campanhas de Saúde Pública (SUCAM)-institutions of the Ministry of Health-to work in the region of the Municipality of Ilhéus, Brazil, participating mainly in the control of malaria and the bubonic plague-important regional endemics at that time.

He was selected to attend the Master’s Course at the School of Public Health in Mexico. After completing this course in the early 1980s, he was invited to work at the headquarters of SUCAM, in Brasília, working in the divisions for the control of yellow fever and dengue and then nationally coordinating the schistosomiasis control^1^. With the extinction of SUCAM, he continued his activities at the National Health Foundation (FUNASA). He also extensively participated in the Polio Elimination Campaign of the National Secretariat for Basic Health Actions by the Ministry of Health, which had a great success in controlling polio in the country. 

He represented Brazil in numerous international meetings in countries in South America and other regions like Mexico, Guatemala, Dominican Republic, Panamá, United States, England, Switzerland, and China.

He retired from FUNASA after making excellent contributions to the control of endemic diseases in the country.

Dr. Ronaldo developed many technical-scientific activities that resulted in the publication of scientific articles, either as an author or as a co-author, among which we highlight:

*An analysis of the impact of the Schistosomiasis Control Programme in Brazil. Memorias do Instituto Oswaldo Cruz, v. 101, 2006.* *La esquistosomiasis mansoni en el Brasil: epidemiologia y control. Revista Salud Pública, BAYER, v. 15, 2000.* *Evolução e situação atual do controle da Esquistossomose no Brasil. Revista da Sociedade Brasileira de Medicina Tropical, v. 27, n.III, 1994* *Criadouros de Aedes aegypti encontrados em alguns bairros da cidade do Rio de Janeiro-RJ/Brasil, em 1984-1985. Cadernos de Saúde Pública (FIOCRUZ), Fiocruz, v. IV, n.3, 1988.* *Aplicação espacial de inseticidas em Saúde Pública. Cadernos de Saúde Pública (FIOCRUZ), FIOCRUZ, v. IV, n.2, 1988.* *Duas ameaças de um mosquito: Febre Amarela e Dengue. Revista a Saúde no Brasil, 1983.* *Investigação comparativa sobre a prevalência de Geohelmintíases em relação com elementos de Saneamento Básico das Habitações, na população menor de 15 anos, na cidade de El Grullo no Estado de Jalisco-México. Revista Baiana de Saúde Pública, Bahia-BA, v. 2, 1982.*

Dr. Ronaldo was President of the Brazilian Association of Sanitarians in the 1990s and fought hard to establish a state career for sanitarians at the federal level. Unfortunately, this objective has not been achieved so far.

In his final days, he left us a great lesson in courage. He calmly rejected any palliative measures such as blood transfusion and hemodialysis, accepting the inexorable evolution of the underlying disease that would lead to his death.
